# Perceptual training to improve hip fracture identification in conventional radiographs

**DOI:** 10.1371/journal.pone.0189192

**Published:** 2017-12-21

**Authors:** Weijia Chen, David HolcDorf, Mark W. McCusker, Frank Gaillard, Piers D. L. Howe

**Affiliations:** 1 School of Psychological Sciences, University of Melbourne, Parkville, Australia; 2 Radiology Department, Royal Melbourne Hospital, Parkville, Australia; 3 Radiology Department, University of Melbourne, Parkville, Australia; Waseda University, JAPAN

## Abstract

Diagnosing certain fractures in conventional radiographs can be a difficult task, usually taking years to master. Typically, students are trained ad-hoc, in a primarily-rule based fashion. Our study investigated whether students can more rapidly learn to diagnose proximal neck of femur fractures via perceptual training, without having to learn an explicit set of rules. One hundred and thirty-nine students with no prior medical or radiology training were shown a sequence of plain film X-ray images of the right hip and for each image were asked to indicate whether a fracture was present. Students were told if they were correct and the location of any fracture, if present. No other feedback was given. The more able students achieved the same level of accuracy as board certified radiologists at identifying hip fractures in less than an hour of training. Surprisingly, perceptual learning was reduced when the training set was constructed to over-represent the types of images participants found more difficult to categorise. Conversely, repeating training images did not reduce post-training performance relative to showing an equivalent number of unique images. Perceptual training is an effective way of helping novices learn to identify hip fractures in X-ray images and should supplement the current education programme for students.

## Introduction

Diagnosing medical imagery is a complex task. To identify abnormalities and reach a correct diagnosis, radiologists must correctly interpret a large number of visual cues. These cues can be masked by the generally noisy background in an X-ray image, variation between patients, slight differences in positioning, or even be mistaken for normal anatomical structures [1]. Residents are typically trained to diagnose conventional radiography in a rule-based manner, usually with no effort made to cluster numerous examples of the same study or provide immediate gold-standard feedback. Given the visual complexity of X-ray images, learning to diagnose radiography in this manner can be difficult. In this study, we investigated how perceptual learning may apply to radiology training and whether students could more rapidly learn to diagnose proximal neck of femur fractures in conventional frontal radiographs of the hip.

Humans can easily make perceptual distinctions that are hard to verbalise. A classic example is sex identification. People usually have no trouble correctly recognising a face as male or female and rely on a large number of visual cues to do so. These cues include distances between key points on the face [2], the luminance contrast between the eyes, lips, and the surrounding skin [3], the shape of facial features [4], and hair cues [5]. Although each of these cues is individually unreliable, people are able to combine them to make rapid and robust decisions [2, 3, 6]. Traditionally, advanced visual recognition skills were assumed to develop only from years of practice because they are difficult to explicitly teach. Recent studies, however, suggest that such skills can in fact be acquired rapidly within days, or even hours, via perceptual training [7].

The term *perceptual training* refers to any training whose goal is to improve perceptual skills, such as the ability to recognise or categorise images. The learning that results from such training is known as *perceptual learning* [1, 8]. Instead of learning to interpret a medical image by following a set of explicit rules, students can instead be trained to visually *recognise* abnormalities. While there have been a large number of perceptual training studies (for a review see [9]), to the best of our knowledge, there have been only three studies that investigated perceptual training in the context of diagnosing medical images, and only one in radiology. Sowden, Davies and Roling [1] trained novices to identify microcalcification clusters in mammograms by presenting them with 60 images, three times a day. Each image contained a microcalcification cluster and the task was to localise it. Immediate feedback was given. An improvement of 17% in localisation accuracy was observed after three days of training. Similarly, Krasne, Hillman, Kellman and Drake [10] trained first and second year medical students in skin histopathology using 261 unique images. The students were required to classify each image as containing pathological or normal histology. The students were immediately informed if they were correct or not. Despite a median training time of only 15 minutes, mean categorisation accuracy increased by approximately 13%. Finally, in a recent study by Xu, Rourke, Robinson and Tanaka [11], naïve undergraduate students were shown 100 images, one at a time, and asked to indicate whether or not the image contained a melanoma. As before, the students were immediately informed if they were correct or not. When subsequently tested on a new set of images, their false alarm rate had decreased by 19% compared to their pre-training performance.

Much of the previous research into perceptual learning has assumed that it is a low-level phenomenon that is achieved by the modification of receptive fields of neurons in early sensory cortical areas that are responsible for the initial encoding of the stimulus [1, 9]. However, accumulating evidence has argued against this viewpoint. In particular, it has been found that perceptual learning will sometimes transfer between retinal locations [7, 12]. This finding is not compatible with the assumption that perceptual learning is achieved by neurons in early sensory cortical areas as these neurons have highly localised receptive fields. It has also been reported that the changes in receptive field structure in early cortical areas are often too small to account for the behavioural changes induced by perceptual learning [13]. More robust receptive field changes seem to occur at later stages of visual processing, for example cortical area V4 [13].

For the purposes of this study, we will assume that perceptual learning is not achieved solely by changing the receptive fields in early cortical areas. Instead, following the lead of Eleanor Gibson [8], we will conceptualise it terms of later cortical areas learning to select the outputs of earlier cortical areas that are most relevant for making the required perceptual distinction. Following Petrov et al., we assume that the selection procedure is not all or nothing but instead occurs by later cortical areas weighting the outputs of early cortical areas when making decisions [14]. For conceptual simplicity, we think of this process as being approximately by a multilayer perceptron [15]. A multilayer perceptron is an abstract representation of the neural system. It is an artificial neural network comprising multiple layers of nodes, each layer typically fully connected to the next one as a directed graph. Each node performs a weighted sum of its inputs and passes this value through a nonlinear activation function to determine its output. The weights therefore determine the degree to which nodes in one layer can affect the activities of nodes in a subsequent layer. Perceptual learning can be achieved by altering these weights so as to select the outputs of the previous layer that are most relevant for making the required perceptual distinction. As such, multilayer perceptrons represent a useful and practical model of perceptual learning [15] and can be used to make novel predictions.

In our study, we investigated perceptual training in an area in which it had not previously been applied, diagnosing radiographs. From the previous literature, it is clear that perceptual learning can improve a participant’s ability to diagnose medical images. However, it is less clear to what extent this improvement is practically useful. For example, could perceptual training be used to train a naïve participant up to the level of a board certified radiologist or would the required amount of training be prohibitive? Our first aim was therefore to quantify the amount of perceptual training required to do achieve this level of performance.

The second aim of our study was to determine the best way to optimise our perceptual training. Multilayer perceptrons generally learn quicker when trained only on the more difficult training images [16]. To the extent that multilayer perceptrons model perceptual learning, it follows that perceptual learning should also occur quicker when humans are exposed only to the more difficult training images. Our second aim was to test this prediction.

Our final aim was to investigate to what extent images can be reused during perceptual learning. It has been shown the repeatedly showing the same training images is an effective way to train multilayer perceptrons [15]. Our third aim was therefore to investigate whether repeating image presentations resulted in further perceptual learning (as opposed to showing the images just once) and, if so, to compare the degree of perceptual learning achieved by repeating images to the degree of perceptual learning achieved by showing an equivalent number of novel images. It is often expensive and time-consuming to obtain medical images. Being able to reuse medical images would therefore make perceptual training more feasible.

## Method

### Participants

The previous study that is most similar to ours suggests that significant effects can be observed with just 10 observers [11]. Because we are in a different area (radiology as opposed to dermatology), as a precaution we used more participants. For the six experiments listed below, in total, 142 novices with no prior knowledge in X-ray film interpretation participated. The number of novices in each experiment is shown in Table 1. They were mostly undergraduate students from The University of Melbourne, aged between 17 and 50 years (*M* age = 23.5 years, *SD* = 5.5 years; 49 men). Additionally, we also evaluated the performance of board certified radiologists and first and second year radiology residents. Unlike the novices, these participants received no training and did not participate in the experiments listed below. The five board certified radiologists were between the ages of 30 and 41 years (*M* age = 35.8 years, *SD* = 5.4 years; 4 men) and had an average experience of 8.5 years. The five 1^st^ and 2^nd^ year residents were between the ages of 25 and 32 years (*M* age = 28.0 years, *SD* = 3.2 years; 3 men) and had an average experience of 1.2 years. All participants had normal or corrected-to-normal visual acuity (at least 20/25; near-field Snellen eye chart) and normal colour vision (Ishihara plates). This study was approved by The University of Melbourne Human Research Ethics Committee (Ethics ID 1545113) and all experiments were performed in accordance with the ethical standards laid down in the amended Declaration of Helsinki [17]. The research ethics committee approved the lack of parent or guardian consent for participants under the age of 18. All participants gave written informed consent. Six experiments were conducted in total, from 19^th^ January to 16^th^ November, 2016. Those in the novice group were paid AU$15/hour for their participation, and in Experiments 2–6 they could additionally earn a bonus of AU$5 if they scored above 85% in the post-training test. Results were analysed using IBM SPSS Statistics 22.

**Table 1:** The number of participants in Experiments 1–6.

Experiment	No. of Participants
1	23
2	25
3	22
4	23
5	24
6	25

### Apparatus and stimuli

The stimuli were cropped hip region images from AP pelvis X-rays selected from the film archive of the Royal Melbourne Hospital of patients who presented to the emergency department with a fracture of head or neck of femur, in all cases confirmed surgically. The images were anonymized and digitised using the FujiFilm Synapse PACS v4.5 software and presented on a black background. Annotations were made using Skitch by Evernote. From each pelvis X-ray each hip region was independently cropped generating two non-overlapping images, one of the left hip and the other of the right hip, one of which would contain a fracture, while the other would not. For the sake of consistency, these images were always presented as the right hip (i.e., any left hip images were flipped so that they looked like a right hip). X-rays were excluded if the fracture occurred in a region with abnormal underlying bone (i.e. pathological fracture due to bone tumourabnormal underlying bones abnormal, such need to include this—I' any metalware (i.e. total hip replacement, dynamic hip screws). X-rays were also excluded if the non-fractured hip exhibited grossly abnormal pathology, such as tumours or Paget disease. Osteoporosis and osteoarthritis in either hip were not considered to be exclusion criteria. Images were also excluded if there was any metalware (i.e. total hip replacement, dynamic hip screws) in either hip.

### Procedure

The stimuli were presented on a personal computer using MATLAB^®^ and the Psychophysics Toolbox [18, 19]subtending an area of 25.8° × 16.2° at the 60 cm viewing distance. They were presented in darkened room similar to the conditions under which radiologists report. A single image was presented on each trial and participants were instructed to indicate whether the X-ray image contained a fracture or not by pressing one of two keys on the keyboard. The image remained on screen until a response was made.

#### Pre-training test

In Experiments 1 and 2, each participant in the novice group was first tested on 20 images to establish their pre-training accuracy. No feedback was given in this stage. There was no pre-training test for Experiments 3–6.

#### Training phase

**Fig 1:**
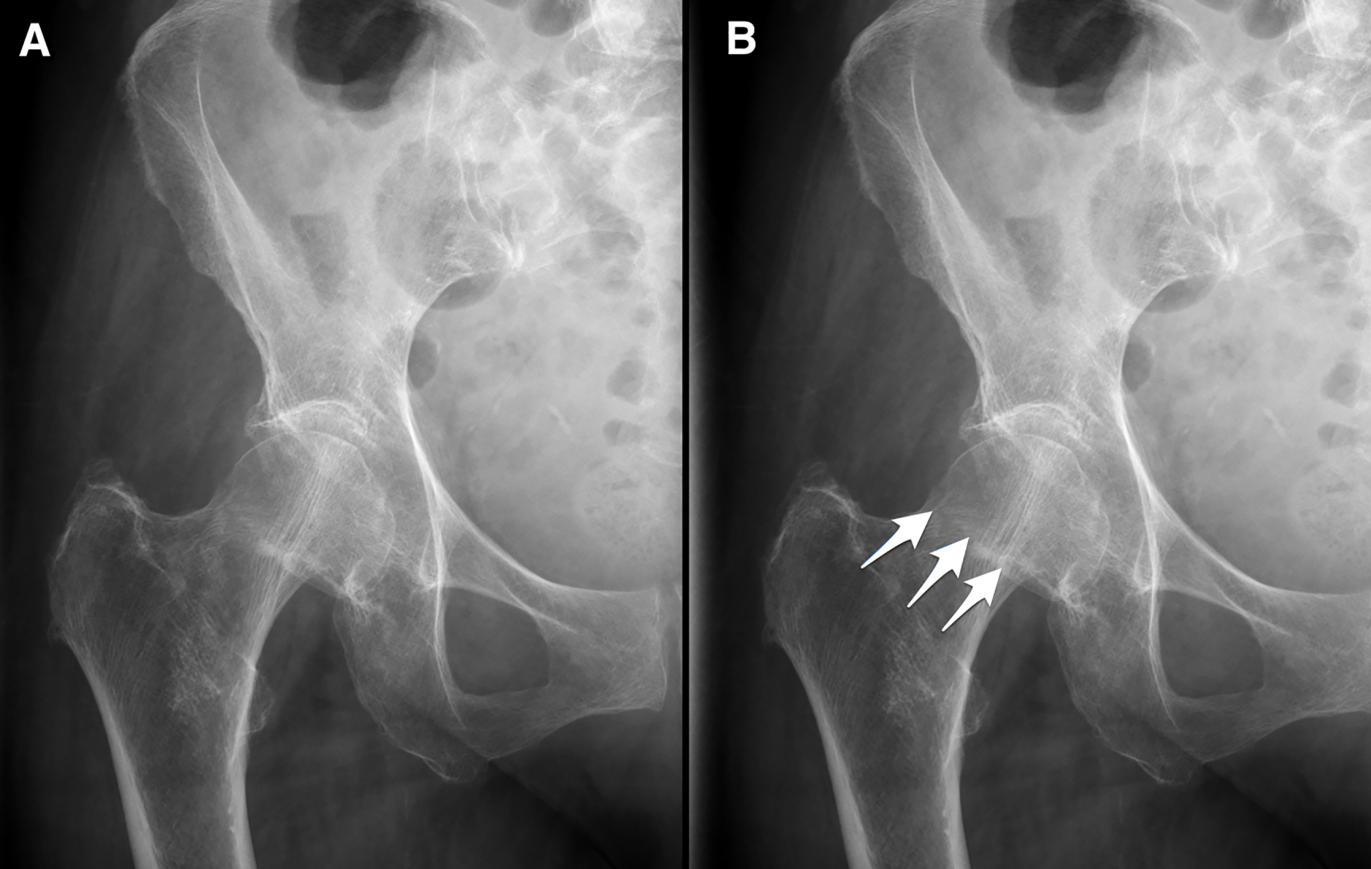
Examples of images shown to participants. **(a)** An example of the image initially shown to a participant. The image is of the right hip and pelvic region of a 76-year-old female. A fracture is visible. **(b)** An example of the feedback the participant would receive if the participant were, in this case, to incorrectly state that there was no fracture.

The training phase was conducted in a single session in Experiments 1–5. In Experiment 1, participants were shown 200 unique images, one at a time, and asked to indicate whether or not the image contained a fracture. Participants received feedback on every trial. Specifically, if they made a mistake, an error message appeared and the same image was shown again, with arrows indicating the location of the fracture, if there was one (Fig 1). Participants earned one point for each correct response and the total score was shown on screen to motivate performance. In Experiment 2, the training procedure was the same but the novices were only trained on the relatively more difficult images based on the performance of the novice group in Experiment 1. This was to test the hypothesis that training solely on the more difficult images would lead to greater perceptual learning. Sixty-two images that had previously been classified correctly 5–75% of the time were selected as training images. We chose a wide accuracy range so that it was difficult but still possible to classify those images correctly. We purposely chose a large range of accuracies to minimise the possibility that we were only training observers to categorise images of a particular difficulty. This training block was presented to participants four times for a total of 248 trials. In Experiment 3, novices viewed a single block of 320 unique images. These images were selected randomly without regard to difficulty level. In Experiment 4, each participant was presented with the same block of 320 images twice; and in Experiment 5 participants viewed a single block of 640 unique images. This was to investigate the effect of repeating the training image set on post-training accuracy. Experiment 6 was conducted over two days. On the first day of training, each participant was presented with 640 randomly chosen X-ray images. They then viewed the same block of training images on the following day. In all six experiments, the target prevalence rate was 50%.

#### Post-training test

After the training session, each participant was tested on a single block of new images to assess their post-training accuracy. No feedback was provided in this test. The post-training test consisted of 180 images in Experiments 1 and 2. This was reduced to 80 images in Experiments 3–6, because novices continued to learn in the post-training test even in the absence of feedback. The training and test images were randomly selected from an image pool without replacement. The board certified radiologists and 1^st^ and 2^nd^ year residents received no training and were tested on 180 images randomly selected from the same image pool as was used for the novices.

## Results

Accuracy was calculated as the sum of hits and correct rejections divided by the total number of trials. The data of nine participants from Experiments 1–5 were excluded from the analysis because their accuracy was at or below the chance level of 50% throughout the experiment, indicating a lack of engagement with the task. Two participants from Experiment 6 were excluded because they did not return on the second day of training.

In addition to the mean post-training accuracies for all novices, we also conducted analysis on the top five novices to explore how training affects the top quartile of our trainees. Because radiologists are selected on merit, we analysed the results of the top five performing novice participants in an attempt to control for ability variations, so as to give a more realistic estimate of the training effects. Participants in the novice group were ranked based on their accuracy in the first half of the post-training test. For each experiment, the top five performing participants were selected based on their responses to the first half the post-training test. We then calculated the mean accuracy of these participants based on their responses to the second half of the post-training test.

The average pre-training accuracy for the entire novice group obtained from Experiments 1 and 2 was 55.9%. This was significantly higher than the guess rate of 50%, *t*(47) = 2.31, *p* = .03. However, all the novices who participated in this study confirmed before commencing the experiment that they had no prior knowledge of X-ray film reading. On average, it took participants 2.77 seconds to read each image in the training phase. The mean reading time for the experts in the test phase was 4.62 seconds per image.

**Fig 2:**
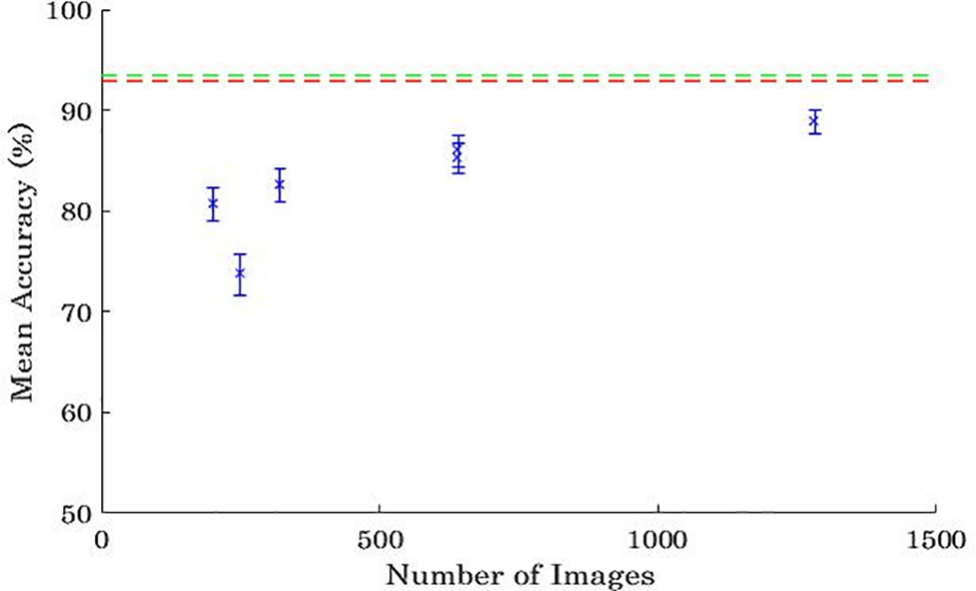
Mean accuracies of the board certified radiologists (dashed green line), radiology residents (dashed red line) and the average of all the undergraduates (blue data points) in Experiments 1–6 plotted against the number of training images viewed in each experiment. Error bars represent standard error of the mean.

**Fig 3:**
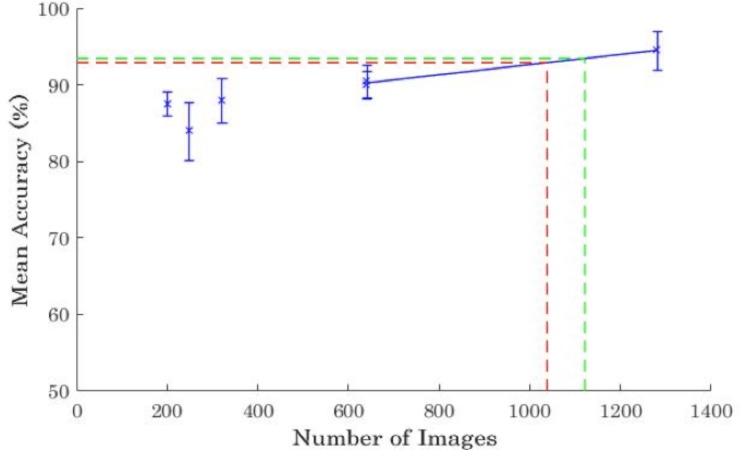
Mean accuracies of the board certified radiologists (dashed green line), radiology residents (dashed red line) and the top five undergraduates (blue data points) in Experiments 1–6 plotted against the number of training images viewed in each experiment. Error bars represent standard error of the mean. The data from Experiments 4–6 was interpolated to predict the number of training images required to bring the accuracies of the top five undergraduates up to the same level as the radiology residents and board certified radiologists.

The mean accuracies for the entire novice group in each experiment are presented in Fig 2. For the sake of comparison, the mean accuracy of the residents is shown by the dashed red lines and the mean accuracy of the board certified radiologists are shown by the dashed green lines. Fig 3 shows the equivalent data for the top five participants for all the experiments. Interpolation was used to determine how many training images are needed to bring the level of the top five novices up to the level of the residents and board certified radiologists. In general, mean post-training accuracy increased as the number of training images increased, with the exception of Experiment 2. *t* tests revealed that the average post-training accuracy for all novices in Experiment 2 was lower than that in Experiment 1, *t*(39) = 2.73, *p* = .01, Cohen’s *d* = .85, despite Experiment 2 utilising more training images than Experiment 1. This shows that training the participants on just the more difficult training images did not improve performance, contrary to what we predicted. Conversely, there was no significant difference in post-training accuracy in Experiments 4 and 5 regardless of whether the results are averaged over all novices (*t*(43) = 0.32, *p* = .75, Cohen’s *d* = .004,) or just over the top five novices (*t*(8) = 0.18, *p* = .86, Cohen’s *d* = .02,). This indicates that showing each image twice did not reduce the effectiveness of the training. As predicted, reusing images is an effective instructional strategy.

As shown by Fig 2, when accuracy is averaged over all novices, the mean accuracy of the novices did not exceed that of the residents or the radiologists, even when trained on 1280 images. While it is possible that further training might have improved performance further, it is unclear whether with sufficiently training could ever be brought up to the standard of residents. The performance of residents and radiologists may in part reflect the considerable amount of rule-based conceptual training they receive, in addition to perceptual training.

Conversely, Fig 3 shows that the mean accuracy of the top five novices exceeded that of the experts. While the underlying learning function for novices is likely to be a power function [20], it can be approximated by a linear function over a sufficiently narrow range. We therefore performed a linear interpolation of the data in Experiments 4–6 and predicted that training novices on 1,037 images could bring the mean accuracy of the top five novices to the same level of residents. To train on this number of images would take approximately 48 minutes. To increase the accuracy of top five novices to the same level of proficiency as board certified radiologists would require 1,121 images and approximately 52 minutes of training.

For conceptual ease, the above analysis was presented in terms of percentage correct. We obtained essentially the same results when we repeated this analysis using a bootstrap comparison [21] of *d’* (S2 File). The advantage of *d’* is that it is a measure of sensitivity that is independent of any response bias the observers may have [22]. In this case, discounting the response bias did not materially affect our results.

## Discussion

The primary aim of this study was to determine how much perceptual training is required to bring naïve observers to the same level of proficiency as board certified radiologists in identifying hip fractures on frontal hip radiographs. It also explored ways to optimise the training procedure, and set a foundation upon which further research can build. For the average undergraduate in this study, training did not improve their accuracy to the same level as the experts. Moreover, the average reading time in our novice group was faster than the average time spent on each image by the experts. This pattern of response fits a classic speed-accuracy trade-off explanation for their performance. Biggs et al. [23] conducted a difficult conjunction search task with professional airport baggage screeners and non-professionals. They found that non-professional searchers terminated searches faster than professionals and that response time was the primary predictor of search accuracy for them. But the more experienced professionals responded slower and had more consistent RTs on every trial, in turn, RT consistency best predicted their performance. On the other hand, our results from the top five novices suggest that under one hour of training with approximately 1,100 images is sufficient to train the top performing undergraduates to the level of accuracy of board certified radiologists. This shows that perceptual training is highly effective and argues for its potential inclusion in the standard radiology curriculum, or at least for modification of existing ad-hoc training to emphasise clustering of study type and need for immediate feedback.

Comparing the findings of Experiments 1 and 2 reveals that training participants solely on the more difficult images can be detrimental to their performance compared to training them on a random sample of images. This was the opposite of what we predicted and may be due to the training sample in Experiment 2 (i.e. a sample containing only the more difficult images) being non-representative of the types of images radiologists generally see. The fact that no difference in post-training accuracy was observed between Experiments 4 and 5 demonstrate that repeating a training image does not necessarily reduce training effectiveness. This was expected and is in broad according with the multilayer perceptron literature where training typically involves repeatedly showing the multilayer perceptrons the same training images [15].

A number of types of learning could have taken place during the training. Novices would have acquired a familiarity with the appearance of a healthy femur and its bone tissues, and would have learnt to search the image for abnormal patterns. Some sensory-learning based enhancements in sensitivity may also have occurred [1]. Unlike Sowden, Davies and Roling [1] we did not attempt to isolate any one type of learning as this was not the focus of this study. Models of medical image perception typically conceptualise the diagnostic process as involving a holistic first glimpse of the image, followed by a serial search process in locating any abnormal patterns and then reaching a decision on the significance of any abnormalities found [1, 24, 25]. Our training addressed only the first two components of these models. Accurate diagnosis of pathological features requires sophisticated understanding of disease pathogenesis for which the traditional, concept-based, analytical, rule-oriented approach remains important [10].

An open question is how generalizable are our findings. Because the images were selected at random, there is no reason to believe that our findings would not generalize to all other radiographs showing right hip fractures. In particular, no attempt was made to locate the fracture (if present) at a particular anatomical location. Similarly, no attempt was made to keep the orientation of the femur at a particular angle nor to keep the scale of the image precisely the same. Therefore, slight changes in these parameters are unlikely to affect our results. We did, however, ensure that all the shown images were of right hips, flipping left hip images to make them look as like right hip images, as needed. Thus, while we would expect most of the training to transfer to left hip images, it is possible that there might be a slight reduction in performance for left hip images, which in practice could be avoided by flipping these images so that they look like right hip images. Of more concern is to what degree our findings would extrapolate to diagnosing fractures of other bones. Presumably, there would be some transfer of learning, but the transfer would not be total. As the results of Experiment 1 and 2 showed, performance is maximised when observers are trained on exactly the sort of images that they are tested on and is reduced if the training set is not representative of the test set. As Experiment 2 demonstrated, constructing the training set to over-represent a subset of images reduces the effectiveness of the perceptual training. That said, some diseases are so rare that showing a few examples will misrepresent their naturally occurring frequency. While this cannot be avoided, our findings suggest that this should be minimised as far as possible.

In conclusion, our study demonstrates that perceptual training is likely to be a highly effective technique for training radiologists in at least some aspects of diagnosis and could be incorporated in the radiology curriculum. It may also be a particularly effective method of training non-radiologists (e.g. reporting radiographers and other physicians) in becoming proficient in a specific type of examination (e.g. chest x-rays for intensivists and emergency physicians, mammography for reporting radiographers). While some rare diseases can easily be over represented by showing just a few examples, as far as possible the training set should accurately represent the sorts of images the radiologists would need to diagnose in practice at the frequencies at which these images would naturally occur. Repeating these images does not reduce the perceptual learning relative to showing an equivalent number of novel images. This reduces the number of images that are required for the perceptual training.

## Supporting information

S1 File Perceptual training dataset. (XLSX)

S2 File Appendix. (DOCX)
