# Supplementary material for: Perceptual training to improve hip fracture identification in conventional radiographs
Source: PLoS One. 2017 Dec 21;12(12):e0189192. doi: 10.1371/journal.pone.0189192 (PMC5739398; doi:10.1371/journal.pone.0189192)
Supplement: S2 File — (DOCX) [file pone.0189192.s002.docx]

**Appendix A**

We ran additional analysis to calculate the signal detection parameters, *d’* as a measure of sensitivity and *c* as a measure of response bias. The results are shown in Fig A1 [22]. Consistent with our finding on the mean post-training accuracy, the mean *d’* for all novices increased with the number of training trials except for Experiment 2. A bootstrap comparison [21] revealed that the *d’* in Experiment 2 was lower than that in Experiment 1 in 99.7% of the paired comparisons. This difference was not significant for the top five novices, where mean *d’* in Experiment 2 was lower than that in Experiment 1 only in 88.5% of the paired comparisons.


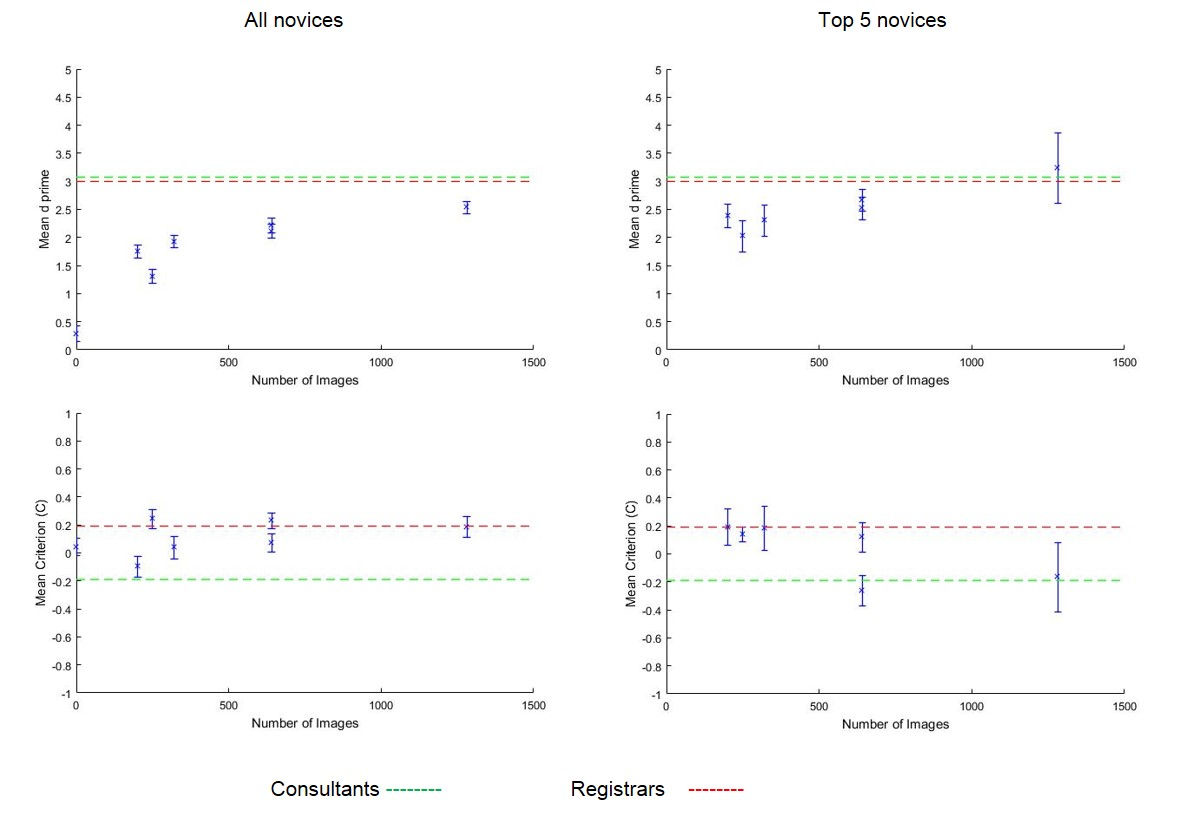


**Fig A1.** The mean signal detection parameters in Experiments 1 – 6 plotted against the number of training trials. Mean *d’* and *c* of board certified radiologists are presented with green dashed lines, and those of the radiology residents are presented with red dashed lines. Except in Experiment 2, mean *d’* improved with more training for all novices (top left) and the top five novices in the second half of their post-training test trials (top right). No systematic change in response bias was observed with increased training in either all novices (bottom left) or the top five novices (bottom right). Error bars represent the standard deviation of the bootstrapped estimate.

No significant difference in *d’* was observed between Experiments 4 and 5, with the *d’* in Experiment 5 being higher than that in Experiment 4 in 71.3% of the bootstrap comparisons for all novices and 31.3% of the comparisons for the top five novices. This was, again, consistent with our results on the mean post-training accuracy.

Mean *d’* of all novices in Experiment 6 was lower than that of the residents in 99.4%, and that of the consultants in 99.9% of the bootstrap comparisons. This finding could explain why mean accuracy for all novices did not reach the same level as the professionals even after 1,200 training images. However, the *d’* of the top 5 novices in Exp. 6 was only lower than that of the residents in 33.0% of the comparisons, and that of the consultants 33.8% in of the comparisons. No significant difference in *d’* was found between the residents and the consultants, with the consultants having a higher *d’* in 77.9% of the bootstrap paired comparisons.

While sensitivity generally improved with increased number of training trials, there was no systematic shifted in criterion with increased training. The consultants were more liberal than the residents in 99.9% of the bootstrap comparisons. Averaged across all novices, the participants in Experiment 2 were more conservative than those in Experiment 1 in 99.98% of the bootstrap comparisons. However, no significant difference in criterion was observed among the top five novices in Experiments 1 and 2, with mean criterion in Experiment 1 being more conservative in 74.3% of the bootstrap comparisons. Interestingly, both the average novice (96.4%) and the top five (98.0%) novices in Experiment 5 (640 unique training images) were significantly more conservative than those in Experiment 4 (320 training images presented twice), despite no difference in mean post-training accuracy between the two experiments for either novice group. The mean criterion in Experiment 6 for all novices was not significantly different from that of the residents, with residents having a more conservative mean criterion in 51.3% of the paired comparisons. But the top five novices had a mean criterion no different from that of the consultants where the latter group was more conservative in 40.1% of the bootstrap comparisons.
